# Meeting of the Michigan Central Dental Association

**Published:** 1872-09

**Authors:** 


### PROCEEDINGS OF SOCIETIES.

[*Continued from Page* 359.]

### Meeting of the Michigan Central Dental Association.

EVENING SESSION.

The committee appointed to examine applicants for admission to the association reported favorably on the admission of Dr. Stuart, of Tecumseh; Welch, of Hudson; Finch and Owen, of Adrian; Nichols, of Ann Arbor; Hunter, of Manchester.

The applicants were ballotted for separately and duly elected.

Drs. Knapp, of Fort Wayne; and Edson, of Toledo, were elected honorary members of the association.

Discussion opened on the subject of Celluloid Base.

Dr. Finch, of Adrian, had been working the celluloid base, and was much pleased with the first he had put up, many of the plates sent out by the company at first had been imperfect, but the company had now remedied the evil, and they were as good as rubber on the average, and the work of putting them up was no more than with rubber or any other material. He used an oil bath, and was much pleased with the base.

Dr. Edson, of Toledo, said that by putting a teaspoonful of chloride of lime in the oil bath, the unpleasant odor of the oil would be removed.

Dr. Haroun, said that by inserting the plaster cast in a solution of boiling borax it would become very hard.

Dr. Edson, had used the celluloid, and preferred it to anything he had seen yet for cheap bases. The oil in the bath should be very hot, and the plate should be left in some ten or fifteen minutes. It was very easily mended, and was not marred in the process.

Dr. Finch, thought there were some cases, perhaps, in which, if he was using rubber, he should prefer it to the celluloid.

Dr. Taft had no personal experience but in the college laboratory they were using it with good success. Dr. Palmer had thought it much better than rubber, but care should be taken to have it sufficiently heated, and the pressure should be applied gradually.

Dr. Taft did not think rubber was fit to be worn in the mouth as a bas plate. The mucous membrane was almost always injured by a rubber plate. He thought this injury more liable from a rubber than from any other plate. The celluloid far preferable to rubber. It is stronger than either gold or rubber. The introduction of rubber and block teeth had prostituted the practice of dentistry, and de stroyed the beauty of dentures.

Discussion closed on this point, and the next topic—The Relative Merits of Tin and Amalgam—should the latter ever be used—was taken up. The President read a paper on this subject, written by Dr. H. White, his partner. The writer preferred gold, but as gold filling could not always be used, amalgam was his next choice; it did good service if properly used. He detailed his mode of using it.

The discussion was participated in by Drs. Taft, Harroun, Robinson, Jackson, Dorrance. The first named gentleman was intensely opposed to the use of any kind or variety of amalgam for filling, and the general opinion seemed to sustain his views.

Shortly before 10 o'clock the meeting adjourned to 8.30, Thursday morning.

THURSDAY MORNING.

The meeting was called to order by the President, the ordinary routine of business was disposed with, and the first topic of discussion was stated, "What are the characteristics of a good border against which to pack gold ?''

Dr. Robinson, said that in confining the rubber dam on the most difficult teeth, it had been his practice to take a piece of spunk cut into a bar, and after winding the silk about the tooth, drawing the piece of spunk to the dam and bind it with the silk, and this gave the best results.

Dr. Dorrance, thought gold could not be packed against a thin edge of enamel. A good border should have a smooth, square edge. It might be of enamel.

Dr. Taft said it was difficult to give a concise answer to the question. If enamel was always of the same texture it would be easier to answer, but it differed in various stages of life, and in various persons. All the circumstances must be taken into consideration in making up the course of treatment in any case. He gave some very useful technical information on the point in question. The aim in filling should be not only to dislodge extraneous substances that were causing decay, but to restore, as far as possible, the form and size of the tooth as it was originally.

"Destroying pulps with carbolic acid," was the next topic in order. Dr. Hawxhurst, of Battle Creek, has used carbolic acid in a number of cases, and had met with good success. He had failed in some instances with the acid, and then used cobalt. He had used the acid in both the crystal form and in the solution.

Dr. Holmes, of Battle Crecek had met with good success in the use of carbolic acid.

Dr. Harroun and Clippinger both used the instrument in destroying the nerve. The latter did not destroy as many nerves as he used to do. Dr. Edson used ether spray in destroying sensibility, and then took out the nerve. Dr. Robinson, preferred to hear how to preserve the nerve, instead of discussing means to destroy it.

Dr. Taft took the same view as did Dr. Robinson. The destruction of the pulp was, in most instances, the ultimate detruction of the tooth. The dentine and the enamel were supported through the pulp, and the removal of this support necessitated the deterioration of the tissues, and the ultimate breaking down of the structure. The idea so prevalent among the profession a short time ago, that every exposed pulp should be destroyed, had its birth in ignorance, and he was glad a change was taking place in this respect. All pulps could not be saved alive, but many could and should be. One great trouble of the profession was that it failed to take into consideration the condition of the patient. Some pulps of strong vitality failed to be killed by applications of carbolic or arsenious acid. The aim of the profession should be to save, and not to destroy.

But if a case arose in which the pulp must be destroyed, he would not use arsenious acid. He would use an instrument. The ether spray was a good agent in destroying sensibility, but he would not use arsenic in devitalizing the pulp.

Dr. Stuart, thought the people should be educated to a care of their teeth. Every dentist should do his share in this good work by occasionally calling the attention of the people to the negligence so prevalent in the care of the teeth. Parents should be educated to a proper care of the teeth of their children.

Dr. Robinson, thought the actual good work done by dentists would have more effect than all the treatises that could be written. The saving of the life of teeth had always been a pet hobby of his. He had been a pioneer in this idea in the profession, and he was pleased to see so many coming ever to his view.

After some desultory discussion not entirely connected with the subject at issue, the next topic was opened. This was—"How shall we treat exposed pulps in the permanent teeth of children under ten years of age."

Dr. Taft said he had frequently treated exposed pulps in the teeth of young children, but they were by no means certain of success. Good results were difficult to arrive at, from various causes. The teeth were not fully developed, children were not patient, and in many cases the vitality was so low that it was impossible to save the teeth.

In answer to various questions, Dr. Taft gave much valuable information relative to the manner in which the teeth drew their nourishment and attained their development. He also reiterated many of his views, as expressed yesterday, relative to the treatment of the pulp.

AFTERNOON SESSION.

Dr. Taft demonstrated the mode of applying the rubber coffer-dam, used in keeping the teeth dry while being filled, and then occupied a short time, at the request of the President, in discussing the code of dental ethics, particularly that section relating to advertising. The Doctor took strong grounds against humbug advertising and empiricism, and recommended the young men of the profession to always do the best they could, always do the very best their judgment dictated for their patients. Then success would be certain.

A vote of thanks was tendered Dr. Taft for his attendance, and the much useful information he had conveyed to the meeting.

Dr. Robinson, spoke strongly in favor of dental associations, and urged the formation of an association which should embrace a part of Michigan and a portion of Ohio. He closed his remarks by moving that the next meeting be held on the third Wednesday of January next, in Jackson, The motion was carried.

Dr. Jackson, suggested that the Association take some steps looking to the establishment of a chair of dentistry in the State University. He thought there was an opportunity to establish such a chair, if the proper influences were brought to bear on the Regents. There could be no question of the good results which would follow from such a course, and he was extremely anxious to see a movement made in that direction.

A letter was read from Dr. C. E. Bärtlett, of Battle Creek, regretting his inability to be present at the meeting of the Association, and expressing a preference for the celluloid, rather than the rubber base.

"The value of the microscope in medicine and dentistry," was stated as the next topic for discussion, and Dr. Hauxhurst, read a paper on the subject, strongly urging the use and benefit of the microscope in the practice of dentistry and medicine.

A vote of thanks to the essayist was passed.

"Dr. Arthur's system of fill ng" was stated to be the next topic of discussion. Dr. Hauxhurst, of Battle Creek, explained the system as laid down in Dr. Arthur's book. Dr. Arthur favored the careful dressing of the proximal surfaces of teeth that were in contact. He then explained at some length the course recommended by Dr. Arthur.

Dr. Robinson, stated that some years ago it was the custom, in the Southern States especially, to clip away the under corners of the teeth, thus affording no place for the lodgment of the food. The practice was adopted because of the bad success which at first attended the filing of teeth. But since filling had become so successful an operation the plan was less in favor.

Dr. Knapp, of Fort Wayne, said that he had heard many expressions, from prominent dentists, relative to Dr. Arthur's method, but he did not think dentists would be prepared to adopt Arthur's plan entirely.

Dr. Knapp, of Adrian, stated that Drs. Arthur and Palmer were entirely opposed in theory. The latter favored the entire restoration of the teeth, the former cut away the teeth as much as possible. He thought Prof. Palmer's method the best.

Dr. Smith, of Jackson, took the same view as did Dr. Knapp; he favored the Palmer method as opposed to the Arthur method.

Dr. Holmes, of Battle Creek, did not desire to see injustice done to Dr. Arthur. He was not prepared to adopt the whole of Arthur's method, but he thought there was much of good in it. He did not agree with Arthur that the dentine would, if polished, make as good a surface as the enamel.

Dr. Dorrance moved that the President authorize a person to receive and read the essays competing for the prizes; that person to read the essays, and to transfer them to an examining committee who should pass on their merits without knowing the authors. The motion was adopted, and Dr. Robinson, of Jackson, was appointed to read the essays.

Dr. Owen, of Adrian, read an essay on, "When and why to extract teeth." He held that no tooth should be extracted if it was possible to save it. He thought many mistakes were made by parents and dentists in the care of desidious teeth. They are often destroyed with the idea of making room for the adult teeth. It was the duty of the profession to instruct parents that childhood's teeth are childhood's servants, and they should not be extracted unless such a course should be absolutely necessary.

A vote of thanks was tendered Dr. Owen, and he was requested to allow his essay to be placed on file.

The assocation then adjourned to meet in Jackson in January next.

